# Evaluating the Impact of Short-Notice Accreditation Assessment on Hospitals’ Patient Safety and Quality Culture: Protocol for a Mixed Methods Study

**DOI:** 10.2196/76945

**Published:** 2026-04-22

**Authors:** Robyn Scanlan, Tracy Flenady, Amy-Louise Byrne, Jenni Judd

**Affiliations:** 1 Reseach Division, Central Queensland University, University Drive, Bundaberg, 4670, Australia, 61 418714569; 2 Central Queensland University, Rockhampton, Australia

**Keywords:** mixed methods, health care quality, health care standards, staff perceptions, organizational culture

## Abstract

**Background:**

Accreditation programs are used by hospitals and health services to be independently reviewed against established safety and quality standards and have been a feature of global health systems for over a century. While evidence that accreditation programs directly improve patient safety and quality outcomes exists, the findings of various researchers remain mixed. Inefficiencies and a culture of “gaming” the system have also been observed, raising questions about the overall effectiveness of accreditation programs and assessment processes. Consequently, exploration of other formats of accreditation assessment, such as short-notice accreditation assessment, has arisen. From July 1, 2023, the Australian Commission on Safety and Quality in Healthcare mandated that Australian public and private hospitals must engage in short-notice accreditation assessment.

**Objective:**

This study aims to explore the impact of short-notice accreditation assessment on hospitals, both in terms of safety and quality indicators, and organizational culture. A mixed methods design will be used to investigate these impacts.

**Methods:**

Quantitative safety and quality indicators will be drawn from a regional health service prior to and following its first short-notice accreditation assessment cycle. From the same site, staff will be invited to complete the Patient Safety Culture Survey and participate in semistructured interviews. Using Schein’s Culture Framework as an organizational culture model, the study will examine observable outcomes (artifacts, behaviors, and indicators) alongside staff perceptions and experiences (norms and values) to form an understanding of underlying assumptions and beliefs about short-notice accreditation assessment processes. Quantitative data will be analyzed through cross-tabulation, trend analysis, and other statistical techniques, while qualitative data will be synthesized to provide a comprehensive understanding.

**Results:**

This protocol outlines the planned evaluation of short-notice accreditation assessment and its influence on patient safety and quality culture within a regional health service. Data collection is underway, with preintervention surveys being completed, and recruitment open for postintervention interviews. The study is expected to generate new knowledge on how this accreditation assessment process affects patient safety and quality culture of a regional and a rural hospital.

**Conclusions:**

The findings will inform health policy on the suitability and long-term viability of short-notice accreditation assessment as an approach to ensuring safe, high-quality health care.

## Introduction

Accreditation programs are the process by which hospitals and health services are assessed against contemporary and evidence-based quality and safety standards [1,2] to ensure minimum standards of care delivery are embedded in the organizational functioning, and that such outcomes are received by the recipients of care [3,4].

Accreditation programs in health care originated in the early 20th century as part of efforts to standardize hospital practices and improve patient care [3,5]. Initially, these processes were voluntary and focused on basic structural and procedural standards, such as sanitation and record-keeping. Over time, accreditation programs evolved into a comprehensive system of external evaluation, incorporating evidence-based standards and performance indicators to assess hospitals’ compliance with best practices [5,6]. Today, achievement of accreditation is widely recognized as a cornerstone of health care quality assurance globally, with most health service organizations (HSOs) viewing it as essential for maintaining public trust, meeting regulatory requirements, and driving continuous improvement [3]. In Australian hospitals, engagement in accreditation programs is now a mandated process for public and private services [7], and failure to meet specified standards can lead to regulatory consequences.

Despite accreditation’s longevity in health care, debates persist about whether accreditation truly enhances patient outcomes or primarily promotes compliance behaviors rather than cultural transformation within organizations [6,8-10].

The strong assumption that accreditation programs hold HSOs to the expected level of care delivery and continually improve quality is supported at some level by research and practice [3,6,8,11], with some comparative studies being inconclusive [6,9,10]. In their Evidence Brief, Deeble Institute for Health Policy Research [9] identifies that research into HSO accreditation is lacking, and, subsequently, the value and impact on quality and safety outcomes are ambiguous [9]. Interestingly, Hussein et al [8] identify that accreditation may support improvement in performance measures, but that there is inconclusive evidence to link accreditation with an improved patient safety culture.

Many studies also demonstrate the unintended consequences of the established assurance processes [12-15]. Significantly, the resource investment into preparing and complying with an accreditation assessment has been reported to create additional burden and costs in engaging in accreditation [15-17]. Additionally, as accreditation outcomes may be publicly available, organizational actions that may pursue a positive result could be prioritized [18] over processes to achieve continuous patient care improvement [17-19]. This can result in inefficiencies in the standard accreditation process [6,19,20], a heavy investment in resources to achieve a successful outcome [18,20], and focus on compliance rather than quality [18,19].

Indeed, contemporary literature increasingly critiques the adequacy of hospital accreditation in capturing the true culture and outcomes of HSOs. While accreditation is widely accepted as the mechanism for quality assurance, its effectiveness in driving meaningful cultural change remains contested. Lewis and Hinchcliff [21] argue that the body of research on accreditation is largely atheoretical and fails to explain how or why accreditation programs influence quality improvement, noting that most studies focus on compliance rather than cultural transformation. Similarly, Husseinet al [8] found that although accreditation programs may positively affect safety culture and process-related performance measures, their impact on patient outcomes such as satisfaction and readmission rates is inconsistent and often negligible. These findings suggest that attainment of accreditation may not be a reliable proxy for assessing the deeper, more nuanced aspects of organizational culture.

Further, a growing concern in the literature is the phenomenon of “accreditation readiness” or “gaming the system,” where health services prepare intensively for scheduled assessments, potentially masking the everyday realities of care delivery. The concept of “Accreditation Theater,” as described by the Accreditation Commission for Health Care [22], highlights how punitive and performative assessment practices can create an illusion of rigor without advancing actual care quality. Tactics used during the accreditation assessment may include, but are not limited to, the distraction of accreditors to divert attention away from potential areas of noncompliance; rostering practices to ensure the most compliant staff are present during the assessment review; cognitive bias creation through relationship development with surveyors; and planning and timetabling ensuring areas of noncompliance are potentially hidden during accreditation reviews [19]. Brubakk, Vist [23] further emphasize that the push for accreditation persists despite scant evidence of its effectiveness, and that most studies fail to account for the context and implementation of accreditation processes, which are critical to understanding their true impact. These critiques underscore the need for accreditation models that move beyond episodic compliance checks and instead foster continuous cultural and quality improvement embedded in everyday practice.

Nonetheless, HSOs generally support the value of accreditation programs, which are seen as essential in driving engagement with and developing collective quality improvement [8,24-26] and improvements in organizational cultures generally [27]. However, inefficiencies in the standard accreditation assessment process have raised questions about the value and effectiveness of a planned and cyclic accreditation procedure [8,28].

To drive a continuous state of accreditation readiness, short-notice or unannounced formats of accreditation assessment have been introduced [28,29]. A short-notice accreditation assessment process provides the HSO with a short period of time (eg 48-hours’ notice) that surveyors will be onsite to undertake an assessment; and an unannounced accreditation assessment process provides no notice to the HSO that surveyors will be onsite to undertake an assessment. Notably, the American health care accreditation body, the Joint Commission, introduced unannounced visits in 2006 with the goal of ensuring quality care at all times, built on the premise that it was the “right thing to do” [30]. More recently, the Danish Institute for Quality and Accreditation in Healthcare undertook a trial of unannounced accreditation surveys [19]. Ehlers et al [19] found that unannounced accreditation surveys were not more effective in detecting quality issues in hospitals; however, the study did not examine the impact of an unannounced model on the safety and quality culture of HSOs. Subsequently, an Australian-first trial of a short-notice accreditation assessment occurred in 2017, resulting in changes to the Australian Health Service Safety and Quality Accreditation Scheme (AHSSQAS) mandating short-notice accreditation assessment processes for all Australian public and private hospitals [7,17,20].

Those Australian HSOs engaged in short-notice accreditation assessment trial argue this type of process ensures assessment results reflect the reality of the HSO safety and quality environment, and embeds a culture of quality and safety functioning within the daily practice, thus improving patient care [20], experience [31], and outcomes [20]. Importantly, short-notice accreditation assessment offers an alternative to traditional accreditation assessment processes, which may reduce opportunities for gaming. Indeed, short-notice or unannounced assessment processes of accreditation have been proposed to minimize the resource investment in preparing for accreditation [7,20], support the reliability of the accreditation outcomes [17,20,28], and reposition the accreditation program as a tool to support HSOs’ quality and safety agenda [20,29].

Despite the change to a new format of accreditation assessment, there is a paucity of evidence that supports the use of a short-notice assessment process [19,28], and little research that explores the impact that this process has on the culture of quality and safety for a health service. Scanlan et al [32] concluded that there is limited evidence reporting on the effectiveness of short-notice accreditation assessment models used by hospitals, and that there are no studies undertaken which seek to understand the impact a short-notice accreditation assessment model can have on patient safety and quality cultures. As such, this study protocol details a mixed methods study which uses Schein’s Culture Framework [33] as a theoretical model within a postpositivism paradigm to evaluate the impact of a short-notice accreditation assessment.

## Methods

### Research Aims

This research aims to understand the impact of short-notice accreditation assessment processes on HSO patient safety and quality culture, and to understand the impact of a short-notice accreditation assessment process from a safety and quality indicator perspective.

### Research Objectives

The research objectives are as follows:

1. Measure and compare HSOs' safety and quality key performance indicators before and after the short-notice accreditation assessment.
2. Evaluate the effectiveness of short-notice accreditation in increasing individual accountability and translating safety and quality standards into clinical practice.
3. Assess the extent to which short-notice accreditation embeds a sustainable safety and quality culture within the organization.

### Theoretical Framework

This study adopts a postpositivist orientation consistent with its mixed methods design and focus on empirical evaluation of organizational outcomes. Postpositivism supports the use of quantitative indicators to examine patterns and relationships in safety and quality performance, while also recognizing the value of qualitative inquiry to explore staff perceptions and experiences that cannot be fully captured through measurement alone.

Schein’s Culture Framework [33] provides a pragmatic structure for integrating these data sources by linking observable organizational artifacts and behaviors with reported norms, values, and assumptions. This approach enables a comprehensive examination of how short-notice accreditation processes may influence patient safety and quality culture without requiring commitment to a single explanatory theory of organizational reality.

Measuring organizational culture is notoriously difficult and complex [34], with agreed indicators lacking [35]. In simplest terms, culture is the pattern of shared basic assumptions within a group [33]. Typically, HSOs focus on the observable and easily measured outcomes to infer the underlying culture of their organization [36,37]. Research into determinants of positive patient safety and quality health outcomes indicates culture is a significant driver [38-42]; however, limited information in triangulating accreditation, health service outcomes, and culture exists [43-46].

No standard tool for measuring organizational culture of complex systems such as HSOs exists; however, the Schein Cultural Framework [33] remains constant in published approaches [47-49]. Cacciattolo [50] synthesized contemporary knowledge and research into applying models for understanding and analyzing organizational culture, finding that 2 main approaches to understanding organizational culture exist: interpretive and structural [50]. The Schein Culture Framework [33] fits within the structural approach [50] and provides a valuable model for understanding a culture’s influence on HSO indicators. Hogan and Coote [51] and Cacciattolo [50] have identified the Framework’s wide acceptance and practical application for understanding and influencing organizational cultural change. While Hogan and Coote [51] have recognized the limited research validating Schein’s Culture Framework [33], they have also demonstrated the value of using the model to build a positive organizational culture [51]. Understanding the determinants of a positive patient safety and quality culture within the health care setting is central to this study, and Schein’s Culture Framework [33] was determined to be beneficial in devising this research approach.

Schein’s Culture Framework [33] is particularly well-suited for evaluating organizational culture in health care settings due to its multilayered approach that captures both visible and invisible dimensions of culture [49]. Compared to other models, Schein’s [33] framework is uniquely positioned to explore organizational-level culture in complex systems like hospitals. Indeed, Schein’s Cultural Framework [33] excels in diagnosing and influencing culture within specific institutions by acknowledging the interplay between structure, meaning, and behavior. Moreover, its compatibility with critical realism, as discussed by Khaddour [52], supports a nuanced understanding of culture that avoids oversimplification and embraces complexity, making it ideal for mixed methods research in health care. This theoretical robustness and practical applicability justify its use in studies aiming to assess the cultural impact of short-notice accreditation processes.

**Figure 1.**
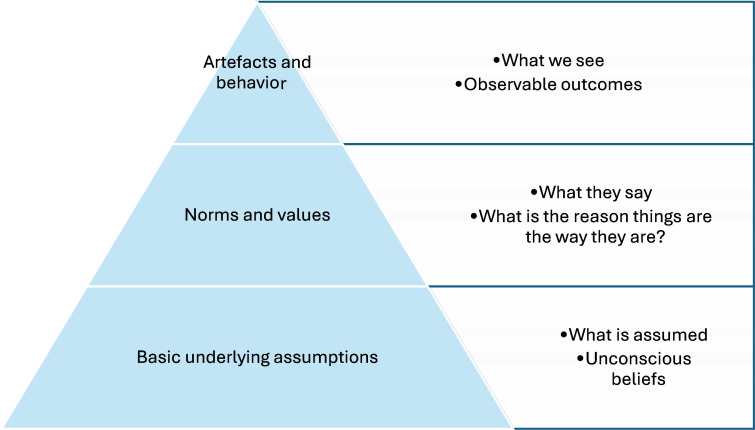
Schein’s [33] culture framework.

Schein [33] proposes a culture framework where understanding the norms, values, and underlying assumptions can be linked to the observable outcomes of organizational indicators (Figure 1).

Using the Schein [33] Culture Framework as a model, the research will consider the effectiveness of a short-notice accreditation assessment process to embed a culture of safety and quality, demonstrated through observable outcomes in organizational indicators. The research will test the underlying but inconclusive belief that accreditation and, subsequently, short-notice accreditation assessment improves patient safety and quality outcomes.

### Design

Considering both organizational culture and impact on quantitative and qualitative indicators lends this research to a mixed methods approach and triangulation of the resultant data through a convergent design process [53]. A mixed methods approach to the research will be used with reporting adhering to the GRAMMS (Good Reporting of A Mixed Methods Study) [54] guidelines.

**Figure 2.**
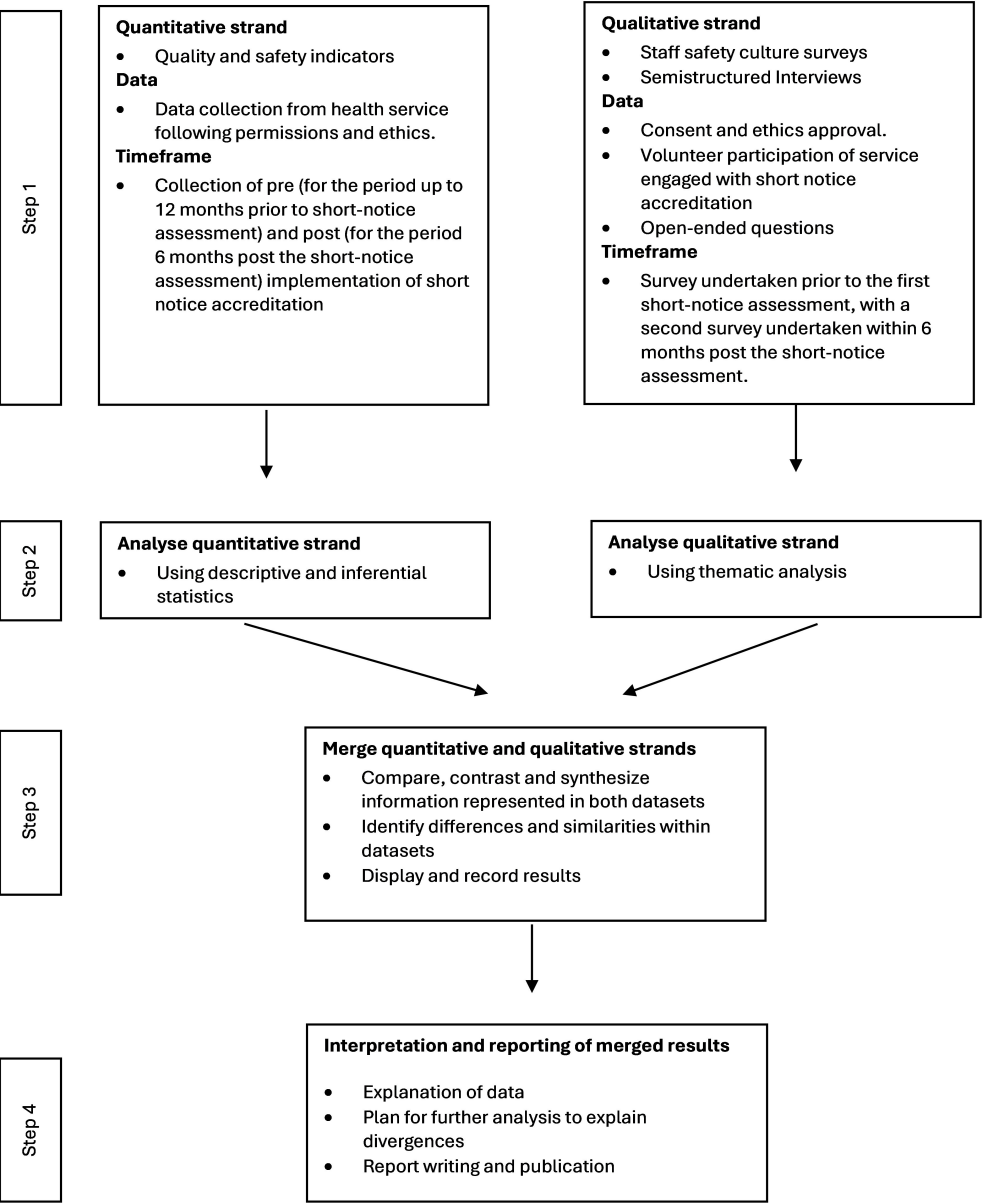
Mixed methods design.

Using a convergent mixed methods design, the research methods and data collection will progress as per the basic flowchart in Figure 2.

(Adapted from Creswell [53]). Hogden et al [55] identified that a mixed methods approach is advocated when evaluating health care safety culture [55]. Their literature review identified that no individual tool could evaluate the safety culture and recommended that a mixed methods assessment of culture as one of the approaches that could be taken [55].

The quantitative data strand will use descriptive and inferential analysis methods. Descriptive statistical methods summarize, describe, and draw conclusions on the population or datasets, while inferential statistical methods use the sample to draw conclusions and make predictions [56]. For the qualitative branch, coding and thematic analysis will be used. Both data strands will be merged to compare, contrast, and synthesize the information, leading to interpretation and reporting, as per Figure 2.

Table 1 illustrates how the anticipated research measures map to Schein’s Culture Framework levels [33].

**Table 1.** Anticipated research measures versus Schein’s [33] culture framework levels.

Schein’s levels	Definition	Anticipated research measures
Artifacts and behaviors	What we see Observable outcomes	Established health service safety and quality indicators
Norms and values	What they say What is the reason why things are the way they are	Staff perceptions and experience
Basic underlying assumptions	What is assumed Unconscious beliefs	Accreditation improves patient safety (not a measure, but an underlying belief or assumption)

### Research Environment

The research will be conducted in one Queensland (Australia) public HSO. The research will include two hospitals: a 331-bed regional hospital and a 36-bed rural hospital. Both hospitals provide various medical services, including emergency, inpatient, outpatient, and allied health services. While the short-notice accreditation format of assessment has been mandated since July 1, 2023, this HSO has yet to engage in their first short-notice accreditation assessment. Including both hospitals from this HSO offers an opportunity to evaluate the applicability of a short-notice accreditation assessment process across various contexts. Importantly, the research environment is shaped not only by the characteristics of the hospitals themselves but also by the external factors such as policy or funding shifts, which may influence organizational culture. By selecting hospitals within the same service area, the researcher can better account for these contextual influences. Interview questions will further explore organizational norms, values, and underlying assumptions about accreditation processes that underpin a patient safety and quality culture.

### Data

#### Overview

A scoping review [32] conducted by the research team identified gaps in understanding the impact of short-notice accreditation. Based on these findings, the study will focus on three components of data collection: (1) safety and quality indicators, (2) patient safety culture surveys, and (3) staff interviews.

**Figure 3.**
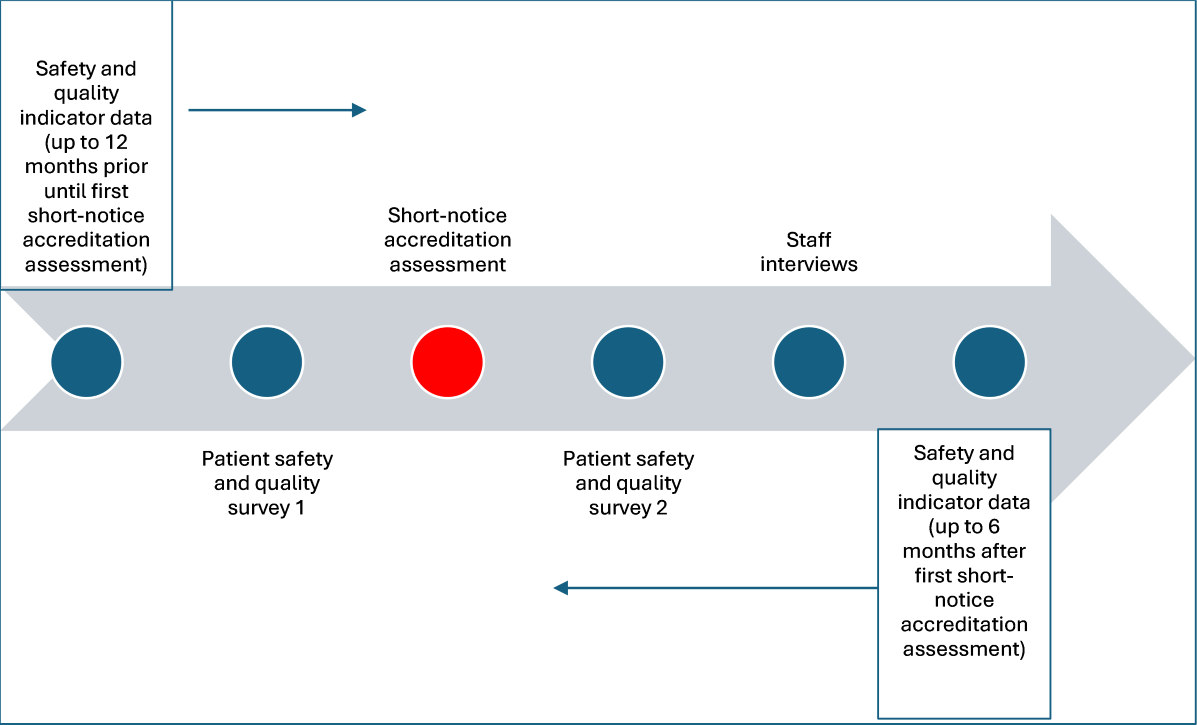
Data collection timeline.

All safety and quality indicators, as well as participants for the surveys and interviews, will be drawn from the regional HSO in which the study is being conducted. Figure 3 provides a graphical representation of the data collection timeline.

Data from all domains will be collected longitudinally. Safety and quality indicators will be examined for the 12 months preceding the HSO’s first short-notice accreditation assessment to establish baseline trends. Because the HSO will not know the exact timing of their assessment, these 12 months of preassessment data will serve as the baseline. Postassessment data will then be collected for 6 months following the first short-notice accreditation assessment, enabling comparison to determine whether the assessment influenced safety and quality indicators. While shorter than the pre-period, the 6-month time frame postassessment is adequate to identify trends pre- and postaccreditation assessment and to inform researchers of future research direction.

Patient safety culture surveys will be administered both before and after the first short-notice accreditation assessment. Staff interviews will be conducted after the accreditation assessment, allowing participants to reflect on and compare their experiences of previous accreditation processes with the new short-notice format.

#### Safety and Quality Indicators

HSOs routinely collect quality and safety indicators as part of their routine governance [37,57]. The indicators are widely recognized as being linked to hospital performance, safety culture, and patient outcomes [36]. Numerous studies have also used clinical quality and safety indicators to evaluate the impact of accreditation processes [19,29,57,58].

In Australia, HSOs establish safety and quality indicators in alignment with the National Safety and Quality Standards [1] which are reliably collected against agreed data specifications. For this study, standard indicators aligned to the National Safety and Quality Standards [59] will be used.

Table 2 presents the indicator population used within the research study.

**Table 2.** Safety and quality indicators.

National safety and quality standard	Process and outcome indicator set	Balancing or linking indicators
Standard 1 – Clinical Governance	Hospital standardized mortality ratio Deaths in low-risk diagnostic related groups Registered quality improvements Severity assessment code 1 clinical incident rates	Readmission rates Unplanned return to theater Length of stay Relative stay index
Standard 2 – Partnering with Consumers	Patient reported experience measures	Readmission rates Unplanned return to theater Length of stay Relative stay index
Standard 3 – Infection Control	Hospital acquired infections	Readmission rates Unplanned return to theater Length of stay Relative stay index
Standard 4 – Medication Safety	Reported medication incidents – severity assessment code 1 and 2 Medication complications	Readmission rates Unplanned return to theater Length of stay Relative stay index
Standard 5 – Comprehensive Care	Pressure injuries, falls, malnutrition, delirium	Readmission rates Unplanned return to theater Length of stay Relative stay index
Standard 6 – Communicating for Safety	Discharge summary completion rates	Readmission rates Unplanned return to theater Length of stay Relative stay index
Standard 7 – Blood Management	Hemovigilance incidents – severity assessment code 1 and 2	Readmission rates Unplanned return to theater Length of stay Relative stay index
Standard 8 – Recognizing and Responding to Acute Deterioration	Deterioration scoring Response escalation Rapid response calls Deterioration patient incidents - severity assessment code 1 and 2 Patient or family escalation of care	Readmission rates Unplanned return to theater Length of stay Relative stay index

This quantitative data will be sourced from health service-specific repositories accessible to the research team under the ethics approval protocol. Indicators will be collected for a period beginning 12 months prior to the HSO’s first short-notice accreditation assessment and extending to 6 months post assessment. This timeframe provides both a preassessment baseline and a postassessment follow-up. In contrast to the traditional 3 to 4-year accreditation cycle, this design allows a timely assessment of the potential influence of the short-notice accreditation process on organizational safety and quality indicators.

#### Patient Safety Culture Surveys

The assessment of a HSO's safety culture is mandated by Australian governing bodies [59]. To support this, the Australian Commission on Safety and Quality in Health Care [60] recently released the Australian Hospital Survey on Patient Safety Culture 2.0 (A-HSOPS 2.0) under a Creative Commons license. The validated tool, specifically designed for use within Australian hospitals, will be used to capture staff perceptions of safety culture within the participating HSO. The survey can be securely administered online, allowing staff to provide responses anonymously.

Participation will be voluntary, with the target sample drawn from the population of staff of the HSO undergoing short-notice accreditation. Based on a 90% CI, the required sample size is 66 clinical staff [61]. As the questionnaire records respondents’ professional role, alignment between the response profile and the overall staffing profile will further support the validity of findings.

To ensure confidentiality, the survey will not collect identifying data beyond: (1) role in the organization, (2) department of employment, (3) years of service in the organization, and (4) gender.

These demographic variables will enable comparison of survey respondents with the overall staff population, while aggregated reporting will minimize any risk of re-identification.

#### Staff Interviews

To supplement the quantitative findings from the HSO quality and safety indicators and safety culture surveys, semistructured staff interviews will be undertaken to provide clarification and contextualization. This qualitative component will help validate and deepen the interpretation of the quantitative data. Semistructured interviews, using open-ended questions, will elicit greater depth and understanding of the subjects’ experience [61]. Participants will be drawn from across the HSO undergoing short-notice accreditation, with representation sought from all organizational levels, including executive, governance, clinical, support, and operational staff. Participation will be voluntary.

Interview guides will be informed by analysis of indicators and survey data with questions designed to explore areas where findings may be limited, unclear, or conflicting (Multimedia Appendix 1). Further, interviews will focus on staff’s perceptions of short-notice accreditation, their experience of organizational culture, and the extent to which these domains may intersect.

### Data Analysis

#### Organizational Quality and Safety Indicators

Data from organizational safety and quality indicators will be reviewed in relation to accreditation processes. Cross-tabulation, trend analysis, and statistical analysis techniques will be applied. Both descriptive and inferential statistical methodologies will be used to provide a comprehensive understanding of indicator trends and their relationship to short-notice accreditation [61].

#### Staff Safety Culture Surveys

Survey responses will generate statistical data reflecting staff’s perceptions of safety culture. Cross-trend analysis and inferential techniques, including ANOVA, will be applied to compare differences across staff groups and over time. As with the indicator data, descriptive and inferential statistics will be used to build a comprehensive understanding of patterns and changes [61]. Data will be exported from Qualtrics for a pre- and poststatistical analysis through time-series comparison.

#### Staff Interviews

Interview transcripts will be analyzed using a qualitative data analysis approach, with coding to identify patterns, themes, and relationships between short-notice accreditation processes and organizational culture. Braun and Clarke [62] 6-phase thematic analysis framework will guide the analysis, ensuring a rigorous and systematic interpretation of staff experiences and perspectives.

### Data Compilation and Report Writing

Data compilation and report writing will occur during steps 3 and 4 of the convergent mixed methods process.

Table 3 provides an overview of data sources aligned with Schein’s [33] organizational culture levels and illustrates how each research artifact addresses the research questions.

**Table 3.** Overview of data compilation.

Schein level	Research question	Research artifact	Population	Analysis
Artifacts and behaviors	What impact does a short-notice accreditation process have on a health service organization’s core safety and quality indicators?	Quantitative analysis of organizational quality and safety indicators	Listed in indicators in Table 1 from a single Hospital and Health Service	Statistical analysis Trend over time-correlated to short-notice accreditation cycles and historical accreditation processes
Norms and values	What impact do accreditation processes have on the perceptions of health service organizational safety and quality culture?	Use of mixed methods Staff Safety Culture Surveys Qualitative Staff Interviews	Representative staff at all levels (ward to executive) Nonclinical, clinical, management, executive from a single Hospital and Health Service	Using mixed methods protocols: Survey statistical analysis Thematic analysis
Basic underlying assumption	What impact does a short-notice accreditation process have on a health service’s organizational safety and quality? How does a short-notice accreditation process support a health service organization’s ability to embed a safety and quality culture?	What impact does a short-notice accreditation process have on a health service’s organizational safety and quality? How does a short-notice accreditation process support a health service organization’s ability to embed a safety and quality culture?	What impact does a short-notice accreditation process have on a health service’s organizational safety and quality? How does a short-notice accreditation process support a health service organization’s ability to embed a safety and quality culture?	Merging of qualitative and quantitative data identifying differences and similarities Interpretation and reporting of merged results to answer the question

Table 3 identifies the primary research question for this project. When the datasets are converged, joint displays and triangulation will occur during interpretation, thus contributing to addressing the primary research question.

The qualitative and quantitative strands of data will then be merged for comparative analysis. This process will enable triangulation of findings, allowing discrepancies and inconsistencies to be interrogated and similarities and differences to be explored. The convergent design ensures that outcomes are interpreted in alignment with Schein’s Culture Framework [33], strengthening the validity of the findings. Where divergences are evident, further analysis will be undertaken to provide an increased understanding of the synthesized results.

### Ethical Considerations

This project has been approved by Queensland Health Human Research Ethics Committee, approval number LNR/2022/QGC/84035 (August 4, 2022), and CQUniversity Human Research Ethics Committee, approval number 0000023861 (September 14, 2022).

## Results

The study is currently collecting the preintervention of patient safety and quality survey data prior to the HSO engaging in their first short-notice accreditation process (Figure 3). Through the survey data collection, the study is also enrolling interested participants for postintervention interviews. Final data and results are expected to be completed 6 months after the short-notice accreditation process. The date of this process is not known to the HSO nor the researchers.

## Discussion

### Anticipated Findings

This study protocol outlines research designed to evaluate the impact of a short-notice accreditation process on the patient safety and quality culture of a health care organization. Existing research in health service accreditation provides limited insight into short-notice accreditation influences organizational culture, and this study aims to contribute to addressing that gap.

In their scoping review, Scanlan et al [32] identified limited and sometimes conflicting evidence on the value of short-notice accreditation processes. The reviewed literature, findings were inconsistent in demonstrating whether accreditation improved safety and quality performance [18,19,27,63]. This lack of consensus highlights the importance of further research to provide robust evidence on the cultural and organizational impacts of short-notice accreditation.

With the recent mandate requiring short-notice accreditation within the Australian accreditation scheme [17], there is a clear imperative to understand the effectiveness of such a format. Grounding this research in Schein’s Culture Framework [33] provides a structured lens through which to examine organizational culture across artifacts and behaviors, norms and values, and basic underlying assumptions. When combined with a mixed methods design, this approach allows for a comprehensive exploration of how short-notice accreditation may shape patient safety and quality culture. Findings from this study have the potential to inform both national and international health policy, while also extending the validation of Schein’s Culture Framework [33] in health care settings.

### Limitations

This study is limited to 2 hospitals within a single regional HSO. While findings will provide valuable insights into the impact of short-notice accreditation in this context, the applicability of results to larger, metropolitan, or differently structured services can only be inferred and will not be directly examined within the scope of this protocol.

An important limitation of this study is the potential influence of external factors on both the organizational culture and the safety and quality indicators being measured. The unpredictable timing of short-notice accreditation assessments means that data collection may coincide with broader systemic changes such as policy reforms, funding adjustments, seasonal fluctuations in patient demand, or workforce transitions. These factors can independently affect staff perceptions, organizational performance, and cultural dynamics, making it difficult to isolate the impact of accreditation alone. For example, staffing shortages or leadership changes during the data collection period may skew survey responses or interview narratives, while external policy shifts could influence indicator trends unrelated to accreditation. Although the study design attempts to mitigate these risks by selecting hospitals within the same HSO, the complexity of health care environments means that such confounding variables cannot be fully controlled. Future research may benefit from multi-site studies or longitudinal designs that better account for these contextual influences.

The staff safety culture survey and interviews also introduce methodological constraints. Survey participation is voluntary and may be subject to low response rates or response bias, with some staff groups under-represented. Interview participants, while purposively sampled to represent all organizational levels, may not reflect the full diversity of staff experiences. In addition, reliance on self-report raises the possibility of social desirability or recall bias. Finally, the study applies Schein’s Culture Framework as its theoretical lens. While this provides a structured approach to examining organizational culture, it may not capture all relevant dimensions that influence how accreditation processes shape safety and quality culture.

### Conclusions

The short-notice accreditation format of assessment has been introduced into the AHSSQAS to strengthen and improve accreditation processes. However, evidence on whether this format enhances patient safety and quality culture remains limited. By applying Schein’s Culture Framework [33] within a mixed methods design, this study seeks to generate new knowledge on the organizational impact of short-notice accreditation. Findings will contribute to understanding how accreditation influences the artifacts, values, and underlying assumptions that shape patient safety and quality culture in HSOs. In doing so, the research aims to inform both policy and practice, nationally and internationally, on the effectiveness of short-notice accreditation as a strategy for improving safety and quality in health care.

## Supplementary material

10.2196/76945 Multimedia Appendix 1 Sample interview questions.

## Abbreviations

A-HSOPS 2.0: Australian Hospital Survey on Patient Safety Culture (version 2.0)
AHSSQAS: Australian Health Service Safety and Quality Accreditation Scheme
GRAMMS: Good Reporting of A Mixed Methods Study
HSO: health service organization
