# Supplementary material for: Evaluating the Impact of Short-Notice Accreditation Assessment on Hospitals’ Patient Safety and Quality Culture: Protocol for a Mixed Methods Study
Source: JMIR Res Protoc. 2026 Apr 22;15:e76945. doi: 10.2196/76945 (PMC13102332; doi:10.2196/76945)
Supplement: Multimedia Appendix 1 [file resprot-v15-e76945-s001.docx]

## Supplementary File 1 – Sample Interview Questions

1. Compared to standard accreditation processes, what is your perception about the effectiveness of a short notice accreditation process?
2. What opportunity does a short notice accreditation process provide for staff engagement?
3. Tell me about your perception of the effect a short notice accreditation process has on patient safety and quality culture in your hospital
4. Tell me about your experience with a short notice accreditation process
5. Can you discuss examples of changes in practice or culture in your hospital, which have occurred as a result of short notice accreditation processes?
